# Scaling up prevention of mother-to-child HIV transmission programs in sub-Saharan African countries: a multilevel assessment of site-, program- and country-level determinants of performance

**DOI:** 10.1186/1471-2458-13-286

**Published:** 2013-04-01

**Authors:** Etienne Audureau, James G Kahn, Marie-Hélène Besson, Joseph Saba, Joël Ladner

**Affiliations:** 1 Biostatistics and Epidemiology Unit, Hôtel Dieu, Assistance Publique Hôpitaux de Paris, Université Paris Descartes, Sorbonne Paris Cité, Paris, France; 2 Philip R. Lee Institute for Health Policy Studies, University of California, San Francisco, USA; 3 Axios International, Paris, France; 4 Epidemiology and Public Health Department, Faculty of Medicine, Rouen University Hospital, Rouen, France

**Keywords:** Prevention of mother to child transmission, Developing countries, Africa, Nevirapine, Program evaluation, Organization and administration of child and maternal health services, Multilevel analysis

## Abstract

**Background:**

Uptake of prevention of mother-to-child HIV transmission (PMTCT) programs remains challenging in sub-Saharan Africa because of multiple barriers operating at the individual or health facility levels. Less is known regarding the influence of program-level and contextual determinants. In this study, we explored the multilevel factors associated with coverage in single-dose nevirapine PMTCT programs.

**Methods:**

We analyzed aggregate routine data collected within the framework of the Viramune^®^ Donation Programme (VDP) from 269 sites in 20 PMTCT programs and 15 sub-Saharan countries from 2002 to 2005. Site performance was measured using a nevirapine coverage ratio (NCR), defined as the reported number of women receiving nevirapine divided by the number of women who should have received nevirapine (observed HIV prevalence x number of women in antenatal care [ANC]). Data on program-level determinants were drawn from the initial application forms, and country-level determinants from the Demographic and Health Surveys (DHS) and the World Bank (World Development Indicators). Multilevel linear mixed models were used to identify independent factors associated with NCR at the site-, program- and country-level.

**Results:**

Of 283,410 pregnant women attending ANC in the included sites, 174,312 women (61.5%) underwent HIV testing after receiving pre-test counselling, of whom 26,700 tested HIV positive (15.3%), and 22,591 were dispensed NVP (84.6%). Site performance was highly heterogeneous between and within programs. Mean NCR by site was 43.8% (interquartile range: 19.1-63.9). Multilevel analysis identified higher HIV prevalence (Beta coefficient: 25.1, 95% confidence interval [CI] 18.7 to 31.6), higher proportion of persons with knowledge of PMTCT (8.3; CI 0.5 to 16.0), higher health expenditure as a proportion of Gross Domestic Product (3.9 per %; CI 2.0 to 5.8) and lower percentage of rural population (-0.7 per %; CI -1.0 to -0.5) as significant country-level predictors of higher NCR at the p<0.05 level. A medium ANC monthly activity (30-100/month) was the only site-level predictor found (-7.6; CI -15.1 to -0.1).

**Conclusions:**

Heterogeneity of nevirapine coverage between sites and programs was high. Multilevel analysis identified several significant contextual determinants, which may warrant additional research to further define important multi-level and potentially modifiable determinants of performance of PMTCT programs.

## Background

Since the HIV epidemic began, children have borne a significant part of the burden of morbidity and mortality. Mother-to-child transmission (MTCT) of HIV, occurring principally during labour, was responsible for approximately 390,000 new infections in 2010 [1]. Of great concern is the situation in sub-Saharan African countries, where the largest share of new HIV infections among children worldwide occurs and where women account for 59% of the adults living with HIV infection. Over the past two decades, improved interventions in industrialized nations have reduced MTCT rates to less than 2% [2,3]. In resource-poor countries, applicable measures to reduce transmission include safer delivery practices, infant feeding counselling and support, and use of antiretroviral (ARV) treatment or MTCT prophylaxis [4]. In this field, the prophylactic administration of single–dose nevirapine (NVP) to mothers and newborns has been a crucial step in the prevention of MTCT (PMTCT), due to its convenient administration, low price and high efficacy [5,6]. Single-dose NVP has therefore gained an important role during the 2000’s and been widely available in low-income countries, largely through donation-based public and private PMTCT programs. Since that early period, the availability of a number of ARV interventions has grown steadily worldwide thanks to ambitious international initiatives [7,8]. Current guidelines have evolved to now recommend the use of more efficient and complex multidrug antiretroviral treatment (ART) regimens for PMTCT [9,10].

Despite considerable progress made thus far, improving prevention program effectiveness in the field still remains a complex challenge, as suggested by discrepancies often observed between anticipated and actual uptake of services [4,11]. In low- and middle-income countries, an estimated 35% of pregnant women were tested for HIV in 2010, and 48% of those living with HIV received the most effective regimens (excluding single-dose NVP) for preventing MTCT [1]. Studies have described a range of potential barriers to program performance, at both the individual level where socioeconomic and psychological factors could play a role [12], and the health facility level where the uninterrupted supply of HIV tests and antiretroviral drugs, the infrastructure quality or the presence of properly trained staff and counselling capabilities are important determinants of effectiveness [13-15]. Yet less is known about the contextual factors that could operate at a broader scale and determine to various extents the performance of preventive or therapeutic programs. In this context, multilevel modeling techniques have proven useful to assess the independent roles and interactions between factors nested at different levels [16], including individual-, site-, program- or country-level characteristics that could partly explain or modulate the effectiveness of PMTCT programs scale-up.

A better knowledge of these multidimensional constraints could help improve final coverage and guarantee program success. In addition, there is still great interest in documenting lessons learned from the implementation of single-dose NVP programs, given probable similarities between such challenges and those that programs based on more complex ARV interventions might encounter. Consequently, the objective of this study was to assess the site-, program- and country-level characteristics associated with NVP coverage levels achieved by PMTCT programs conducted in sub-Saharan African countries.

## Methods

### Data source

We used aggregate monitoring and evaluation data collected within the framework of the Viramune^®^ Donation Programme (VDP). Viramune^®^ (nevirapine) has been offered to developing countries since 2000 by Boehringer Ingelheim. Axios International has been responsible for the management of NVP distribution and monitoring of participating PMTCT programs. All sub-Saharan African countries were eligible for participation in VDP. The nevirapine donation was available to governments directly, as well as Non Governmental Organisations (NGOs), international organisations and public and private health care providers with government approval.

Among VDP participating programs (45 programs including 1,776 sites in 23 countries at the time of the study), we selected those with data available by site or single-site programs. During the study period (January 2002 to December 2005), a total of 269 sites were included, participating in 20 PMTCT programs in 15 sub-Saharan countries (Benin, DR Congo, Côte d'Ivoire, Gabon, Ghana, Kenya, Lesotho, Malawi, Nigeria, Rwanda, South Africa, Tanzania, Togo, Uganda and Zambia). No statistically significant differences between selected and not-selected VDP programs were identified with respect to their main characteristics, including institution status, mean number of sites, mean ANC per program per month and mean HIV prevalence (data not shown; p>0.05).

### Primary outcome

To assess the overall performance of PMTCT sites, a coverage indicator was calculated. The NVP coverage ratio (NCR) was defined as the [observed number of women receiving NVP] divided by [the expected number of women who should have received NVP]. This denominator was calculated as the number of women attending antenatal care (ANC) visits multiplied by the observed HIV seroprevalence at each site (number of HIV-positive women/ number of HIV-tested women).

### Site-level data

Following acceptance to VDP, participating institutions were required to submit a progress report every six to 12 months with the following data for each site: number of women registered in antenatal care, number of HIV-tested and HIV-positive women and number of women receiving NVP tablets. These data did not permit assessment of the number of newborn NVP doses provided. The mean NCR over the period of follow-up was calculated for each site from all available progress reports. Predictors assessed at the site level included the site-observed HIV prevalence, the site activity (number of women receiving ANC), and the type of health facility (primary or secondary/tertiary hospital level).

### Program- and country-level data

Inclusion in VDP required programs to submit a descriptive application form and receive approval by a body of independent experts. We collected the following data in application forms: type of responsible institution or organisation (public/private) and number of sites included.

Variables collected at the national level were drawn from the Demographic and Health Surveys (DHS) [17] and the World Bank’s World Development Indicators [18], including: HIV prevalence (among 15–49 year-old persons), AIDS knowledge (percentage having heard of AIDS, comprehensive knowledge [4 components] and knowledge of PMTCT), HIV-related stigma (percentage of respondents with accepting attitudes towards persons living with AIDS), HIV testing coverage (percentage of respondents tested for HIV and having received their results in the last 12 months), GDP per capita (Gross Domestic Product, $), total healthcare expenditure (% of GDP), and rural population (% of total population). We gathered publicly available indicators at the national level, because the mean number of sites per sub-national area defined by DHS data was too low for meaningful statistical analysis (<5 sites per region).

### Statistical methods

Descriptive statistics were computed for the characteristics of the PMTCT programs and are provided as medians (Interquartile Range [IQR]) for quantitative variables and as percentages for qualitative variables. NCR dispersion across sites was assessed by using univariate boxplots. Non-parametric Spearman correlation rank tests (r_s_) were used to assess univariate correlation of NCR with intermediate process indicators (women tested rate, women treated rate).

Multilevel linear mixed models were used in univariate and multivariate analyses to identify factors associated with NCR, accounting for the hierarchical clustering of sites (level 1) nested in programs (level 2) within countries (level 3). The following multivariate modeling strategy was applied: predictors associated with NCR at the p<0.2 level in univariate analysis were entered sequentially, starting from level 1 to level 2 and 3, using a backward stepwise method at each level. Beta coefficients are given for each predictor variable, indicating the adjusted difference in NCR (0–100) between categories for qualitative variables or per 1-point difference in predictor value for continuous variables. Rural population proportion and total healthcare expenditure group-level predictors were modeled continuously after checking for linearity of the regression coefficients. The initial variance partition between levels was explored in an empty (*null*) model without any predictor. The proportional change in the variance (PCV) was then computed in the final model for each level: PCV was defined as the proportion of variance “explained” from the variance initially estimated in the *null*-model. A two-tailed P-value of less than 0.05 was considered to be significant. All statistical analyses were performed using Stata, version 11.0 (StataCorp, College Station, TX, USA).

### Role of funding source

Boehringer Ingelheim had no role in study design, data collection, data analysis, data interpretation, or manuscript development. The corresponding author had full access to all data in the study and had final responsibility for the decision to submit for publication. All data analyses were conducted under the authorization from Boehringer Ingelheim.

## Results

### Sample description

Table 1 summarizes the main characteristics of the 20 programs and 269 sites included in the study. Overall, cumulative follow-up was 2374 months (median: 9.1 months/program) and 283,410 pregnant women attended ANC services in the sites included over the study period. Fifty-five percent of the programs were conducted by private institutions or NGOs. The median number of sites included per program was 3.5 and the majority were primary health facilities (69%).

**Table 1:** Main characteristics of the PMTCT programs and sites included

		**Number**
**Programs (n=20)**		
Median follow-up length in months (IQR*)		9 (5 – 16)
Institution responsible		
Public	Programs	9 (45%)
	Sites	113 (42%)
Private/NGO**	Programs	11 (55%)
	Sites	156 (58%)
Number of sites per program		
	Median (IQR)	3.5 (1–14)
	Mean (min-max)	13.5 (1–80)
African region		
Eastern Africa	Programs	4
	Sites	89
	Expected HIV prevalence	6.7
Western Africa	Programs	6
	Sites	62
	Expected HIV prevalence	4.4
Central Africa	Programs	6
	Sites	51
	Expected HIV prevalence	2.7
Southern Africa	Programs	4
	Sites	67
	Expected HIV prevalence	16.0
**Sites (n=269)**		
Mean monthly ANC*** activity, # of women (IQR)		116 (58 – 175)
Type of health facility		
Primary care clinic		185 (69%)
General or University Hospital		84 (31%)

* IQR: Interquartile range.

** *Non governmental organization.*

*** *Antenatal Care.*

### Global nevirapine coverage results

**Figure 1:**
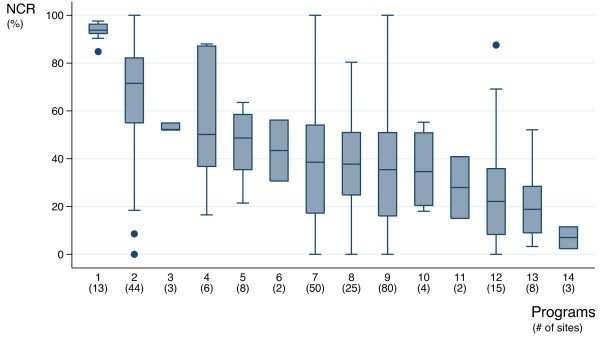
**Site performance dispersion by PMTCT program: Nevirapine Coverage Ratios (NCR).** Boxplots are displayed for programs including at least two sites (n=14) as follows: the median is represented by a horizontal line within each box, which represents the interquartile range (IQR) comprising data between the 25th and 75th percentiles. Whiskers extend to the 1.5*IQR beyond each box and circles represent outliers.

Of the 283,410 pregnant women attending antenatal care, 174,312 women (61.5%) were offered, accepted and underwent HIV testing after receiving pre-test counselling, of whom 26,700 (15.3%) tested HIV positive. After post-counselling, 22,591 women (84.6%) were eventually dispensed NVP. Using these figures, the global NCR was 52.0%. Considering the performance rates per site, the mean testing coverage was 61.5% (IQR 42.2 - 83.8), the mean treatment rate was 70.2% (IQR 50.0 - 98.6) while the mean NCR per site was 43.8% (IQR 19.1 – 63.9). NCR differed greatly across sites ranging from 0.0 to 1.0, and across programs (0.07 to 0.94) as shown in Figure 1. NCR was positively correlated with the women tested rate (r_s_=0.65, p=0.01) and women treated rate (r_s_=0.62, p=0.01).

### Nevirapine coverage determinants

Results from univariate multilevel analysis of PMTCT performance by site-, program- and country-level predictors are shown in Table 2. No significant difference was observed between primary care clinics and general or university hospitals, after accounting for the hierarchical structure of the data through the use of multilevel mixed models. The ANC activity level was correlated with NCR, though in a U-shape, showing lower results in sites with a medium number of women in care (30–100 women/month). Performance results for programs directed by public institutions did not differ significantly from those directed by private ones. Size of program (number of sites) was not significantly associated with better results in testing, treatment or NVP coverage rates, though larger programs had a higher NCR point estimate. Significantly higher performance ratios were achieved by sites from southern Africa (67.9%) and by sites in countries with the highest HIV prevalence, testing coverage and percentage of population with knowledge about PMTCT.

**Table 2:** Univariate multilevel analysis of PMTCT performance by site-, program- and contextual-level predictors: testing, treating and nevirapine coverage ratio (NCR)

	**Number of sites**	**Tested women percentage**	**p-value**	**Treated women percentage**	**p-value**	**NCR**	**p-value**
***Site characteristics***							
Type of health facility							
Primary care clinic	185	61.6	0.79	73.8	0.47	46.8	0.53
General or University Hospital	84	61.2		62.4		37.4	
Activity (antenatal care)							
< 30 / month	90	61.6	**0.01**	73.7	0.06	45.5	**0.05**
30 - 100 / month	84	56.7		63.5		35.8	
>100 / month	95	65.6		72.8		49.4	
***Program characteristics***							
Type of institution responsible							
Public	113	62.5	0.81	66.4	0.28	42.8	0.47
Private/NGO	156	60.8		72.9		44.6	
Number of sites							
<Median (1–3)	16	56.4	0.27	61.9	0.92	35.3	0.38
>Median (4–80)	253	61.8		70.7		44.4	
***Country-level characteristics***							
African region							
South	67	81.2	0.23	82.2	**0.05**	67.9	**0.03**
West	62	52.0		67.9		35.7	
Centre	51	65.6		53.8		33.8	
East	89	50.9		72.2		37.1	
Prevalence of HIV (% of 15-49y)							
<Median (1.2-5.4)	110	57.8	0.32	61.1	**0.002**	34.4	**0.01**
>Median (6.4-23.4)	159	64.0		76.5		50.4	
% Tested for HIV							
<Median (2.8-6.3)	95	54.7	0.22	66.4	0.14	35.7	**0.05**
>Median (7.1-19.6)	170	65.8		72.9		48.9	
% Have heard of AIDS							
<Median (91.8-96.9)	101	59.1	0.79	70.6	0.98	42.8	0.92
>Median (98.8-99.9)	165	63.6		70.8		45.2	
% Accepting (stigma)							
<Median (5.1-29.3)	131	62.8	0.66	70.8	0.31	45.0	0.26
>Median (30.2-48.5)	134	60.9		70.4		43.3	
% With knowledge about PMTCT							
<Median (2.7-33.1)	121	53.6	0.09	67.2	0.14	34.8	**0.01**
>Median (33.3-66.2)	144	68.8		73.5		52.0	
% With comprehensive knowledge about AIDS							
<Median (18.7-30.5)	89	53.4	0.17	66.7	0.54	35.0	0.12
>Median (36.5-55.3)	163	63.6		70.7		45.2	
Rural population (% of total population)							
<Median (17.1-65.0)	122	67.3	0.26	76.2	0.44	53.4	0.18
>Median (68.4-87.6)	147	57.3		66.8		37.2	
Healthcare expenditure (% of GDP*)							
<Median (3.0-6.7)	171	50.9	**<10**^**-4**^	70.5	0.92	36.4	0.10
>Median (7.0-10.0)	98	80.0		69.7		56.9	
GDP per capita ($)							
<Median (144.6-615.8)	141	57.1	0.58	66.7	0.80	36.9	0.75
>Median (1295.3-13338.8))	128	66.4		74.1		51.5	

*Gross Domestic Product.

Multivariate analysis using linear mixed models (Table 3) identified at the site-level a moderate ANC activity (30-100/month) as a negative predictor of NVP coverage (NCR -7.6 when compared to the reference lowest activity category; p=0.05). At the country-level, HIV prevalence (NCR +25.1 for the highest prevalence category; p<0.0001), the proportion of persons with knowledge of PMTCT (NCR +8.3 for the highest knowledge category; p<0.0001) and health expenditure as proportion of total GDP (NCR +3.9 per % of increasing GDP; p<0.0001) were significantly and positively associated with higher NVP coverage, whereas the percentage of rural population was negatively associated with NCR (NCR -0.74 per % of increasing rural population; p<0.0001). There was no significant association found in multivariate models between NCR and the proportion of persons with comprehensive knowledge about AIDS, testing coverage or the African region.

**Table 3:** Multivariate multilevel analysis of PMTCT performance by site-, program- and contextual-level predictors: nevirapine coverage ratio (NCR)

		**β Coefficient**	**p-value**	**(95% CI)**
***Site characteristics***				
	Activity (antenatal care)			
	< 30 / month	*0 (ref)*	-	
	30 - 100 / month	-7.6	0.05	(-15.1 ; -0.06)
	>100 / month	-2.3	0.59	(-10.7 ; 6.1)
***Country-level characteristics***				
	Prevalence of HIV (% of population ages 15–49)			
	<Median (1.2-5.4)	*0 (ref)*	-	
	>Median (6.4-23.4)	25.1	<10^-4^	(18.7 ; 31.6)
	% With knowledge about PMTCT			
	<Median (2.7-33.1)	*0 (ref)*	-	
	>Median (33.3-66.2)	8.3	0.04	(0.53 ; 16.0)
	Health expenditure (% of GDP*)	3.9	<10^-4^	(2.0 ; 5.8)
	Rural population (% of total population)	-0.74	<10^-4^	(-1.0 ; -0.46)

*Gross Domestic Product.

Of the initial total variance of NCR measured in the *null*-model including no site- or program/country-level predictors, 58.5% was found at the site-level and 41.5% at the program/country level (2.7% [program-level] + 38.8% [country-level]). To determine the proportion of variance explained by the predictors retained in the final model, we computed the PCV for each level: PCV was 99.9% for the program/country level whereas only 0.22% of the site-level variance was explained by the final model.

## Discussion

In this study, we analysed aggregate data from 283,410 pregnant women attending 269 PMTCT sites. We identified several factors operating at the country-level that significantly influenced nevirapine coverage in women, including PMTCT knowledge, HIV prevalence, rural population proportion and proportion of GDP dedicated to healthcare expenditure. These factors were independent of site-level factors, such as the site activity or the type of health facility, and program-level factors, such as the status of the institution or the program burden measured as the number of sites included. To our knowledge, this study is the first to explore the contextual determinants of PMTCT program performance within the formalized framework of multilevel modeling. The contextual predictors identified explained almost 100% of the initial variance at the program/country level. Our data also showed a huge heterogeneity of performance between sites within the same programs.

Among 269 PMTCT sites, we found a nevirapine coverage ratio of only 44%. Our result is only somewhat more optimistic than a study by Temmerman et al. reporting a NVP administration rate of 20%, using a similar whole-ANC-population-based calculation process [19]. The take-up testing rate by site was 61.5%, while other studies reported testing rates ranging from 21% to 95% [20-24]. This variability is echoed in our data, since women tested percentages differed across programs from 7% to 97%. Test acceptability after pre-test counselling is variable but overall high ranging from 72% to 97%, as indicated in several previous reports [19,25-27]. In regards to NVP provision to HIV positive pregnant women by site, our finding of 70.2% is consistent with other previous reports indicating rates from 56% to 94% [19,21,23,24,26-31].

Specific PMTCT and general comprehensive knowledge about AIDS were significantly associated with higher nevirapine coverage, whereas general awareness about AIDS – as measured by the proportion of persons who had heard of AIDS – and accepting attitudes or stigma were not significantly linked to NCR. This is consistent with previous reports showing knowledge as an important factor of success in antiretroviral adherence [32] or breastfeeding practices [33]. Above all, this result is encouraging to programs aiming to increase knowledge about PMTCT and AIDS, which is a potentially modifiable contextual determinant. The absence of a significant result for stigma in our study requires cautious interpretation since methods for quantifying stigma are neither straightforward nor consensual. Beyond the difficult medical prognosis, knowledge of HIV positive status is of great social consequence, and stigma and violence are still actual threats for African women [34-36]. Other factors must be taken into consideration for improving HIV test acceptance, such as educational background, familial environment and most importantly male-spousal involvement. Disclosure of HIV status remains problematic [37] and a woman's perception of her husband’s approval of testing plays a crucial role in this context [38].

The national prevalence of HIV remained significant after accounting for knowledge about AIDS and healthcare expenditure. In this context, the interpretation of this indicator might be of interest because it likely encapsulates several other contextual features (e.g. testing coverage, which lost its statistical significance in multivariate analysis) or other factors not assessed in our analysis. Thus, we may interpret national HIV prevalence as an awareness and readiness marker indicative of both population and health services.

Rural population as a percent of total population was negatively associated with NCR. This result is consistent with the loss to follow-up of pregnant women [21], as maternal NVP coverage has been reported to be associated with the number of ANC visits [39]. The quality of follow-up remains a challenge for rural and remote facilities [40]. Interestingly, positive results can been achieved by providing antiretroviral prophylaxis for mother and infant early in pregnancy [12,41] or by offering labor ward-based services for PMTCT [42]. Beyond single-dose nevirapine regimens, rural primary health facilities likely face other difficulties as well, such as the capacity to determine the CD4 cell count required to implement more efficacious antiretroviral regimens [43].

National health expenditure as a proportion of GDP was an independent and positive predictor of nevirapine coverage, probably suggesting an overall positive impact of increased investment in the organization and infrastructure of health services. This finding may confirm the importance of overall health system strength to ensure effective PMTCT services in the field, where performance is notably linked to sites’ infrastructure and testing capacities [13,14] as illustrated in our study by the strong association found between this indicator and the testing rate by site.

At the site-level, the highest and lowest nevirapine coverage results could be observed within the same program, emphasizing challenges to the on-the-ground feasibility of PMTCT programs. Beyond infrastructure and testing capacities, lack of sufficient and well trained staff have been suggested to explain some of the discrepancies observed between sites. Integration of voluntary counselling and testing (VCT) services in pre-existing ANC settings represents an undeniable additional workload for in-place health workers. At the same time and for economic reasons, human resources have not always risen adequately [19,20,25]. Efficient counselling activity requires trained staff fulfilling specific capabilities [25,44]. Test acceptability and women’s satisfaction are moreover strongly related to the capacity of PMTCT services to provide good quality counselling and follow-up care to pregnant women [45]. In our study, a moderate ANC activity (30–100 pregnant women attending ANC services per month) was associated with lower nevirapine coverage ratios. This finding could illustrate the negative effect of increased workload in medium size facilities, where more difficulties may have been encountered to adjust to the increased resource requirements for PMTCT.

We consider the generalizability of our findings in the context of global PMTCT programs. We included VDP programs with data available by site or one-site programs, which raises the question of the representativeness of the sites included, both in regard to the whole VDP program and to other existing PMTCT programs. We did not identify any significant difference between included and not-included VDP programs regarding their main characteristics, which suggests limited selection bias within the VDP program itself. As for the other PMTCT programs, our program panel cannot be considered as fully representative of all programs, implemented either within the same countries or elsewhere in sub-Saharan Africa and/or based on different antiretroviral drugs and regimen designs. However, our analysis was conducted on a range of sites and programs, including primary or secondary/tertiary health facilities with various ANC activities within programs from several different sub-Saharan African regions, thereby supporting the external validity of our results. Secondly, a possible selection bias cannot be excluded in regards to the pregnant women included in this study, since only women actually attending ANC services were considered. Finally, our findings stem from an early era in large scale implementation of PMTCT services, when antiretroviral interventions were based on much simpler regimens than the multi-drug regimens currently recommended [9,10]. However, we believe that most of the factors identified in the present work as determinants of NVP coverage are likely to apply to current antiretroviral interventions as well. Barriers such as a limited access to services in remote rural areas or the influential role of population knowledge about PMTCT still have resonance today. Thus there is still great interest in documenting such obstacles, especially because they were encountered with an intervention as simple as single-dose NVP and may also extrapolate to the much more complex interventions of today. More generally, our results also highlight the potential interest in promoting the use of multilevel analyses when assessing factors affecting program scale-up, be it in the framework of PMTCT or other public health domains. Multilevel approaches are useful for researchers and can bring relevant information to policy makers by weighing the relative impact of site-, program- or contextual-level predictors of successful implementation of health programs.

Our study has several limitations that should be noted. In particular, we were not able to control whether a pregnant woman actually took the NVP dose she was dispensed in PMTCT services. Specifically, we lacked the ability to directly measure the drug compounds contained in cord blood samples, which is an accurate and useful means to counter the limited quality of reported medical data when assessing program coverage [39]. NCR tends to overestimate overall PMTCT coverage, since it does not account for women without access to PMTCT services. We believe NCR can still yield at a glance a direct indicator of the process quality of a PMTCT program or site, encompassing VCT and final NVP administration. Further, we were not able to use DHS sub-national data for this study, given low sample size by area unit (mean number of sites by region <5). Yet, even considering large entities such as countries, a high between-country heterogeneity was observed with respect to contextual factors and NVP coverage, indicating that country-level indicators could still represent relevant variables to assess the ‘average’ contextual environment of the sites within countries. Finally, our results should be interpreted cautiously since we lacked complementary data to describe health facilities (e.g. staff training), as illustrated by the low percentage of variance explained at the site-level in multilevel analysis, or programs (e.g. availability of adherence support services). It is also likely that some relationships identified between NCR and country-level factors, such as the proportion of rural population or healthcare expenditure, may partially but not entirely be explained by uncontrolled site-level factors (i.e. rural/urban facility, quality of infrastructure) that were not available in our dataset.

## Conclusion

In conclusion, we observed a wide range of coverage outcomes among PMTCT sites and programs. To explain these discrepancies, we identified several country-level factors, including knowledge about PMTCT and AIDS or the proportion of GDP allocated to healthcare, adjusting for a variety of site- and program-level characteristics. Our findings illustrate the potential role of surrounding contextual and global health system features, in addition to local characteristics of sites. While qualitative and quantitative research is still needed at the site level, the overall understanding of factors associated with program performance would benefit from further research that attempts to identify more distant but important and potentially modifiable determinants of success.

## Abbreviations

ANC: Antenatal care; DHS: Demographic and Health Surveys; GDP: Gross Domestic Product; HIV: Human Immunodeficiency Virus; IQR: Interquartile Range; NCR: Nevirapine Coverage Ratio; NGO: Non Governmental Organization; NVP: Nevirapine; PCV: Proportional Change in Variance; PMTCT: Prevention of Mother-To-Child Transmission of HIV; VDP: Viramune Donation Programme.

## Competing interests

The authors declare that they have no competing interests.

## Authors’ contributions

All authors participated in the study conception and design. MHB, JS and JL participated in the data acquisition and extraction. EA and JL performed the statistical analysis and interpretation of data. EA made the drafting. All authors participated in the critical revision of the manuscript and have read and approved the final manuscript.

## Pre-publication history

The pre-publication history for this paper can be accessed here:

http://www.biomedcentral.com/1471-2458/13/286/prepub

## References

[B1] WHO, UNAIDS, UNICEF Global HIV/AIDS response - Epidemic update and health sector progress towards Universal Access – Progress Report 2011 2011

[B2] Townsend CL Cortina-Borja M Peckham CS de Ruiter A Lyall H Tookey PA Low rates of mother-to-child transmission of HIV following effective pregnancy interventions in the United Kingdom and Ireland, 2000–2006 AIDS 2008 22 8 973 981 10.1097/QAD.0b013e3282f9b67a 18453857

[B3] Haile-Selassie H Townsend C Tookey P Use of neonatal post-exposure prophylaxis for prevention of mother-to-child HIV transmission in the UK and Ireland, 2001–2008 HIV Med 2011 12 7 422 427 10.1111/j.1468-1293.2010.00902.x 21251184

[B4] Dabis F Ekpini ER HIV-1/AIDS and maternal and child health in Africa Lancet 2002 359 9323 2097 2104 10.1016/S0140-6736(02)08909-2 12086778

[B5] Guay LA Musoke P Fleming T Bagenda D Allen M Nakabiito C Sherman J Bakaki P Ducar C Deseyve M Intrapartum and neonatal single-dose nevirapine compared with zidovudine for prevention of mother-to-child transmission of HIV-1 in Kampala, Uganda: HIVNET 012 randomised trial Lancet 1999 354 9181 795 802 10.1016/S0140-6736(99)80008-7 10485720

[B6] De Cock KM Fowler MG Mercier E de Vincenzi I Saba J Hoff E Alnwick DJ Rogers M Shaffer N Prevention of mother-to-child HIV transmission in resource-poor countries: translating research into policy and practice JAMA 2000 283 9 1175 1182 10.1001/jama.283.9.1175 10703780

[B7] Global Fund Annual Report 2011 2011 Geneva, Switzerland: The Global Fund to Fight AIDS, Tuberculosis and Malaria

[B8] Office of U.S. Global AIDS Coordinator and the Bureau of Public Affairs USSD The U.S. President’s Emergency Plan for AIDS Relief: Eighth Annual Report to Congress on PEPFARhttp://www.pepfar.gov/documents/organization/188019.pdf [Accessed 10 Feb 2013]23559679

[B9] WHO Antiretroviral drugs for treating pregnant women and preventing HIV infection in infants: recommendations for a public health approach – 2010 version 2010 Geneva, Switzerland: World Health Organization 26180894

[B10] WHO Use of antiretroviral drugs for treating pregnant women and preventing HIV infection in infants. Programmatic update 2012 Geneva, Switzerland: World Health Organization 26180894

[B11] Paintsil E Andiman WA Update on successes and challenges regarding mother-to-child transmission of HIV Curr Opin Pediatr 2009 21 1 94 101 10.1097/MOP.0b013e32831ec353 19242245 PMC2650837

[B12] Kuonza LR Tshuma CD Shambira GN Tshimanga M Non-adherence to the single dose nevirapine regimen for the prevention of mother-to-child transmission of HIV in Bindura town, Zimbabwe: a cross-sectional analytic study BMC Public Health 2010 10 218 10.1186/1471-2458-10-218 20426830 PMC2873585

[B13] Ekouevi DK Stringer E Coetzee D Tih P Creek T Stinson K Westfall AO Welty T Chintu N Chi BH Health facility characteristics and their relationship to coverage of PMTCT of HIV services across four African countries: the PEARL study PLoS One 2012 7 1 e29823 10.1371/journal.pone.0029823 22276130 PMC3262794

[B14] Zolfo M De Weggheleire A Schouten E Lynen L Time for "test and treat" in prevention of mother-to-child transmission programs in low- and middle-income countries J Acquir Immune Defic Syndr 2010 55 3 287 289 10.1097/QAI.0b013e3181eef3da 20714271

[B15] Stringer EM Ekouevi DK Coetzee D Tih PM Creek TL Stinson K Giganti MJ Welty TK Chintu N Chi BH Coverage of nevirapine-based services to prevent mother-to-child HIV transmission in 4 African countries JAMA 2010 304 3 293 302 10.1001/jama.2010.990 20639563

[B16] Nash D Wu Y Elul B Hoos D El Sadr W Program-level and contextual-level determinants of low-median CD4+ cell count in cohorts of persons initiating ART in eight sub-Saharan African countries AIDS 2011 25 12 1523 1533 10.1097/QAD.0b013e32834811b2 21750418 PMC3422866

[B17] Measure DHS Demographic and Health Surveys http://www.measuredhs.com[Accessed 10 Feb 2013]

[B18] World Bank World Development Indicators 2013 http://data.worldbank.org/data-catalog/world-development-indicators [Accessed 10 Feb 2013]

[B19] Temmerman M Quaghebeur A Mwanyumba F Mandaliya K Mother-to-child HIV transmission in resource poor settings: how to improve coverage? AIDS 2003 17 8 1239 1242 10.1097/00002030-200305230-00016 12819526

[B20] Rakgoasi SD HIV counselling and testing of pregnant women attending antenatal clinics in Botswana, 2001 J Health Popul Nutr 2005 23 1 58 65 15884753

[B21] Manzi M Zachariah R Teck R Buhendwa L Kazima J Bakali E Firmenich P Humblet P High acceptability of voluntary counselling and HIV-testing but unacceptable loss to follow up in a prevention of mother-to-child HIV transmission programme in rural Malawi: scaling-up requires a different way of acting Trop Med Int Health 2005 10 12 1242 1250 10.1111/j.1365-3156.2005.01526.x 16359404

[B22] Perez F Orne-Gliemann J Mukotekwa T Miller A Glenshaw M Mahomva A Dabis F Prevention of mother to child transmission of HIV: evaluation of a pilot programme in a district hospital in rural Zimbabwe BMJ 2004 329 7475 1147 1150 10.1136/bmj.329.7475.1147 15539670 PMC527692

[B23] Doherty TM McCoy D Donohue S Health system constraints to optimal coverage of the prevention of mother-to-child HIV transmission programme in South Africa: lessons from the implementation of the national pilot programme Afr Health Sci 2005 5 3 213 218 16245991 10.5555/afhs.2005.5.3.213 PMC1831931

[B24] Rispel LC Peltzer K Phaswana-Mafuya N Metcalf CA Treger L Assessing missed opportunities for the prevention of mother-to-child HIV transmission in an Eastern Cape local service area S Afr Med J 2009 99 3 174 179 19563095

[B25] Chama CM Audu BM Kyari O Prevention of mother-to-child transmission of HIV at Maiduguri Nigeria J Obstet Gynaecol 2004 24 3 266 269 10.1080/01443610410001660760 15203622

[B26] Stringer EM Sinkala M Stringer JS Mzyece E Makuka I Goldenberg RL Kwape P Chilufya M Vermund SH Prevention of mother-to-child transmission of HIV in Africa: successes and challenges in scaling-up a nevirapine-based program in Lusaka Zambia Aids 2003 17 9 1377 1382 10.1097/01.aids.0000060395.18106.80 PMC2745990 12799559

[B27] Spensley A Sripipatana T Turner AN Hoblitzelle C Robinson J Wilfert C Preventing mother-to-child transmission of HIV in resource-limited settings: the Elizabeth Glaser Pediatric AIDS Foundation experience Am J Public Health 2009 99 4 631 637 10.2105/AJPH.2007.114421 18703458 PMC2661479

[B28] Harvey KM Figueroa JP Tomlinson J Gebre Y Forbes S Toyloy T Thompson T Thompson K An assessment of mother-to-child HIV transmission prevention in 16 pilot antenatal clinics in Jamaica West Indian Med J 2004 53 5 293 296 15675493

[B29] Albrecht S Semrau K Kasonde P Sinkala M Kankasa C Vwalika C Aldrovandi GM Thea DM Kuhn L Predictors of nonadherence to single-dose nevirapine therapy for the prevention of mother-to-child HIV transmission J Acquir Immune Defic Syndr 2006 41 1 114 118 10.1097/01.qai.0000179425.27036.d7 16340483 PMC1855628

[B30] van't Hoog AH Mbori-Ngacha DA Marum LH Otieno JA Misore AO Nganga LW Decock KM Preventing mother-to-child transmission of HIV in Western Kenya: operational issues J Acquir Immune Defic Syndr 2005 40 3 344 349 10.1097/01.qai.0000160712.86580.ff 16249710

[B31] Ginsburg AS Hoblitzelle CW Sripipatana TL Wilfert CM Provision of care following prevention of mother-to-child HIV transmission services in resource-limited settings AIDS 2007 21 18 2529 2532 10.1097/QAD.0b013e3282f155f4 18025890

[B32] Mepham S Zondi Z Mbuyazi A Mkhwanazi N Newell ML Challenges in PMTCT antiretroviral adherence in northern KwaZulu-Natal South Africa AIDS Care 2011 23 6 741 747 10.1080/09540121.2010.516341 21293987

[B33] Maman S Cathcart R Burkhardt G Omba S Thompson D Behets F The infant feeding choices and experiences of women living with HIV in Kinshasa Democratic Republic of Congo AIDS Care 2012 24 2 259 265 21780955 10.1080/09540121.2011.597708

[B34] Temmerman M Ndinya-Achola J Ambani J Piot P The right not to know HIV test results Lancet 1995 345 969 970 10.1016/S0140-6736(95)90707-6 7619122

[B35] Peltzer K Sikwane E Majaja M Factors associated with short-course antiretroviral prophylaxis (dual therapy) adherence for PMTCT in Nkangala district South Africa Acta Paediatr 2011 100 9 1253 1257 10.1111/j.1651-2227.2011.02253.x 21366691

[B36] Okonkwo KC Reich K Alabi AI Umeike N Nachman SA An evaluation of awareness: attitudes and beliefs of pregnant Nigerian women toward voluntary counseling and testing for HIV AIDS Patient Care STDS 2007 21 4 252 260 10.1089/apc.2006.0065 17461720

[B37] Hardon A Vernooij E Bongololo-Mbera G Cherutich P Desclaux A Kyaddondo D Ky-Zerbo O Neuman M Wanyenze R Obermeyer C Women's views on consent, counseling and confidentiality in PMTCT: a mixed-methods study in four African countries BMC Public Health 2012 12 26 10.1186/1471-2458-12-26 22236097 PMC3295711

[B38] Bajunirwe F Muzoora M Barriers to the implementation of programs for the prevention of mother-to-child transmission of HIV: a cross-sectional survey in rural and urban Uganda AIDS Res Ther 2005 2 10 10.1186/1742-6405-2-10 16255776 PMC1277814

[B39] Coffie PA Kanhon SK Toure H Ettiegne-Traore V Stringer E Stringer JS Dabis F Ekouevi DK Nevirapine for the prevention of mother-to-child transmission of HIV: a nation-wide coverage survey in Cote d'Ivoire J Acquir Immune Defic Syndr 2011 57 Suppl 1 S3 8 21857283 10.1097/QAI.0b013e31821ea539

[B40] Perez F Mukotekwa T Miller A Orne-Gliemann J Glenshaw M Chitsike I Dabis F Implementing a rural programme of prevention of mother-to-child transmission of HIV in Zimbabwe: first 18 months of experience Trop Med Int Health 2004 9 7 774 783 10.1111/j.1365-3156.2004.01264.x 15228487

[B41] Sripipatana T Spensley A Miller A McIntyre J Sangiwa G Sawe F Jones D Wilfert CM Site-specific interventions to improve prevention of mother-to-child transmission of human immunodeficiency virus programs in less developed settings Am J Obstet Gynecol 2007 197 3 Suppl S107 112 17825641 10.1016/j.ajog.2007.03.069

[B42] Megazzini KM Sinkala M Vermund SH Redden DT Krebs DW Acosta EP Mwanza J Goldenberg RL Chintu N Bulterys M A cluster-randomized trial of enhanced labor ward-based PMTCT services to increase nevirapine coverage in Lusaka Aids 2010 24 3 447 455 10.1097/QAD.0b013e328334b285 19926959

[B43] Mandala J Torpey K Kasonde P Kabaso M Dirks R Suzuki C Thompson C Sangiwa G Mukadi YD Prevention of mother-to-child transmission of HIV in Zambia: implementing efficacious ARV regimens in primary health centers BMC Public Health 2009 9 314 10.1186/1471-2458-9-314 19712454 PMC2739530

[B44] Kanshana S Simonds RJ National program for preventing mother-child HIV transmission in Thailand: successful implementation and lessons learned AIDS 2002 16 7 953 959 10.1097/00002030-200205030-00001 11953461

[B45] Hardon AP Oosterhoff P Imelda JD Anh NT Hidayana I Preventing mother-to-child transmission of HIV in Vietnam and Indonesia: diverging care dynamics Soc Sci Med 2009 69 6 838 845 10.1016/j.socscimed.2009.05.043 19576671

